# A more physiological feeding process in ICU: Intermittent infusion with semi-solid nutrients (CONSORT-compliant)

**DOI:** 10.1097/MD.0000000000012173

**Published:** 2018-09-07

**Authors:** Kongmiao Lu, Fei Zeng, Yi Li, Cheng Chen, Man Huang

**Affiliations:** General ICU, Second Affiliated Hospital, Zhejiang University School of Medicine, Hangzhou, China.

**Keywords:** critical illness, enteral nutrition, feeding intolerance, glycemic variability, pepsin, semi-solid nutrients

## Abstract

**Introduction and objectives:**

The goal of this study is to determine whether the application of semi-solid nutrients could increase the efficiency of the enteral nutrition (EN), which was measured daily by administered volume of nutrition/prescribed volume of nutrition.

**Methods:**

A total of 28 subjects were finally enrolled in the study and randomized to receive either intermittent feeding (IF) or intermittent feeding with semi-solid nutrients (IS). Three major parameters concerning EN were evaluated in this study: the daily dosage prescribed by doctor, the actual dosage received by subjects, and the acute complications such as diarrhea, vomiting, regurgitation, bowel distension, and lung infection.

**Results:**

There were no statistical differences in NRS-2002, and acute gastrointestinal injury between both groups. The IS group (0.98 ± 0.06, *P* < .01) could receive higher percentage of daily prescribed calories compared to IF (0.73 ± 0.15). The total caloric intake during the first 3 days was higher in IS (2589.29 ± 844.02 vs. 1685.71 ± 388.00, *P* < .01). The incidence of feeding intolerance (FI) was lower in the IS group (2/14) compared with IF (8/14). However, semi-solid nutrients did not decrease the length of stay, lung infection, or 30-day mortality. Similarly, there was no difference in glycemic variability and stress hyperglycemia.

**Conclusions:**

In our cohort of critically ill subjects, the efficiency of the EN was increased by IS, which might be related to the improvement of FI (NCT03017079).

## 1 Introduction

Enteral nutrition (EN) therapy is an essential part in critically ill patients and can be administered on a continuous or intermittent basis, but there is no consensus on which should be adopted. Continuous feeding is thought to be better tolerated by patients with limited absorptive gut surface area or gastrointestinal dysfunction, but is associated with more tube clogging. It also requires the subject to be attached to an infusion pump for significant periods of time.^[1]^ Intermittent infusion mimics a more physiologic feeding process that allows greater subject mobility. Patients may also reach target enteral calories earlier^[2]^ and decrease length of stay (LOS) and mortality.^[3]^ However, previous studies had reported intermittent infusion was associated with higher complication rates such as diarrhea and regurgitation.^[4]^

Increased EN solution viscosity may prevent aspiration and reflux. High-viscosity liquid meals could decrease the incidence of aspiration in dementia and Parkinson patients,^[5]^ but the study had a small sample size.

Aspiration was a common phenomenon in critically ill patients, but the recessive aspiration was difficult to be found. Previous study found that pepsin was an efficient marker to reflex the reflux that the sensitivity >78.5% with the specificity not <65%.^[6,7]^

In this study, whether semi-solid nutrients can decrease feeding intolerance (FI) and increase the efficiency of the EN was observed. The efficiency of the EN was measured daily by volume ratio (%). VR (%) = (administered volume of nutrition / prescribed volume of nutrition) × 100.

## 2 Methods

### 2.1 Subjects and settings

We performed a prospective, single-blinding, randomized pilot study involving subjects at a large teaching hospital in Hangzhou of ZheJiang province. Ethical approval was granted by Institutional Review Board ([2016] Ethics review research NO. 71) and the study protocol was registered at http://www.clinicaltrials.gov (NCT03017079). Only subjects of brain or spinal cord injury, older than 14 years, with informed consent, were eligible for inclusion. Group assignment was determined by sequentially numbered, sealed opaque envelopes, followed by subject randomization to receive either intermittent feeding (IF) protocol or intermittent feeding with semi-solid nutrients (IS) protocol and only those who continued taking EN by nasogastric tube <72 hours were retained in the final analytic cohort. Subjects who received EN <72 hours, received EN before ICU admission, had acute pulmonary infection, had history of gastrointestinal surgery, or had contraindications of EN such as intestinal obstruction (mechanical or paralytic ileus) were excluded from the study.

### 2.2 Clinical management

Our standard protocol also coupled a mandatory ICU doctor who had nutrition qualification at initiation of EN. The first assessment was typically completed in the first 24 hours and a formal reassessment was performed every 2 to 3 days while the subjects were in the ICU. If there were no absolute contraindications, EN was initiated within 48 to 72 hours of admission to the ICU. The intermittent protocol involved initial feeds of 100 to 300 mL every 4 to 8 hours. This was repeated twice and, if tolerated, they were advanced by 100 to 300 mL every 1 to 2 days to the volume goal. Each intermittent feeding was delivered via an enteral feeding pump during a 30- to 60-minute period of time. The IS only increased the semi-solid agent before the EN application within 1 hour, which is low methoxy pectin gel and water-soluble dietary fiber form apple and citrus peel by binding to calcium ions in EN to increase the viscosity, but do not change any chemical properties of EN.

Samples of the posterior pharyngeal or end trachea were collected in 30 mL standard tubes containing citric acid to preserve the action of any pepsin present for each subject at 1 hour post lunch in 2 to 3 days after inclusion. Pepsin analysis was performed by an investigator who was blinded to the clinical data. The collection tube was centrifuged and the supernatant was collected which mixed with migration buffer solution to the pepsin detecting device.

### 2.3 Clinical data collection

Additional data collected for analysis included ICU admission diagnosis, age, sex, variables to calculate the Acute Physiology and Chronic Health Evaluation (APACHE II) score, Sequential Organ Failure Assessment Score (SOFA Score), incidence of acute ICU complications (lung infection), LOS in the ICU, hospital LOS, 30-day mortality. Data on protein and energy intake were recorded daily throughout the ICU admission for the first 3 days unless there was FI that one of the following symptom happened, such as diarrhea, vomiting, regurgitation, bowel distension, and gastric residual volume (GRV) >200 mL.

### 2.4 Statistical analysis

Data were expressed as means ± standard deviations (SD) for quantitative variables and proportions for categorical and binary variables. Student *t* test was used for statistical comparison between groups for continuous variables, general linear model for repeated measurement data, Fisher exact tests for categorical variables, and Mann-Whitney *U* test for ordinal variables. All tests for statistical significance were determined using an alpha level of 0.05. All analyses were performed using SPSS 17.0 (SPSS Inc, Chicago, IL).

## 3 Results

**Figure 1:**
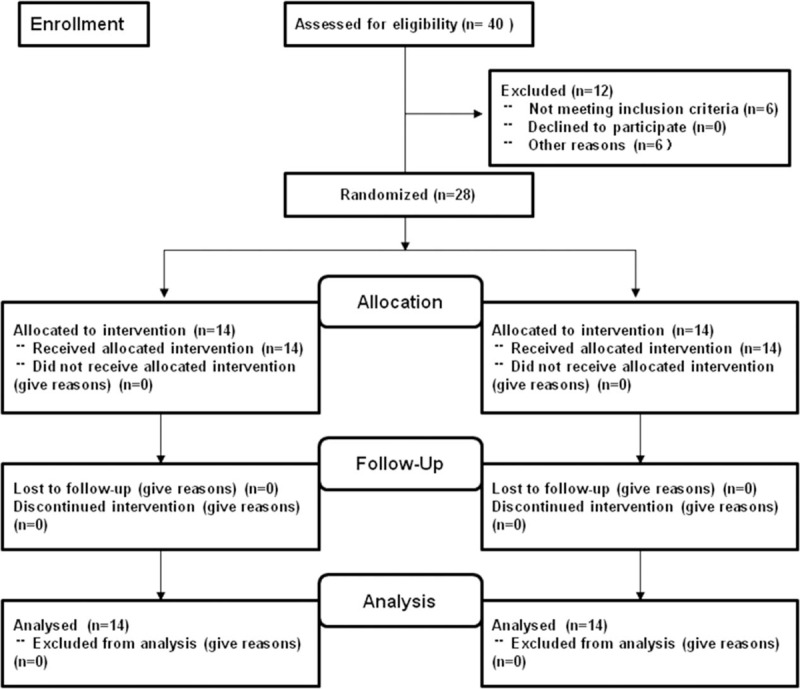
Subjects flow diagram. Figures show flow of study subjects used in overall study (n = 40) and the number of each subgroup. IF = bolus intermittent feeding, IS = bolus intermittent feeding with semi-solid nutrients.

Baseline characteristics for the IF and IS group are presented in Table 1. A total of 40 subjects were recruited, but only 28 subjects were finally enrolled in the study between June 2016 and January 2017 in Figure 1. There were no statistical differences in age, sex, APACHE II scores, SOFA scores, GCS scores, NRS-2002, and AGI per subject.

**Table 1:**
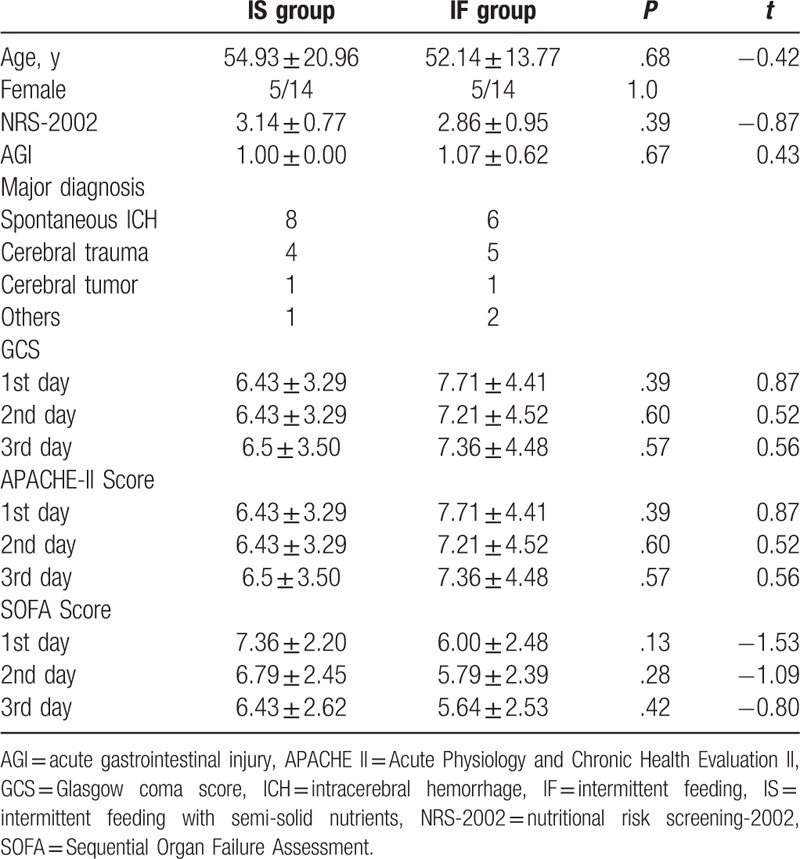
Demographics characteristic.

Except for 3 days caloric intake, the efficiency of the EN and the incidence of FI, there were no differences between both groups in 3 days caloric target and protein intake in Table 2. Subjects feeding with semi-solid nutrients had higher 3 days’ caloric intake (2589.29 ± 844.02 kcal vs. 1685.71 ± 388.00 kcal, *P* < .01) and the percentage of VR (0.98 ± 0.06 vs. 0.73 ± 0.15, *P* < .01) compared to their counterpart. Similarly, the incidence of FI was decreased in IS group of 2/14, compared with 8/14 in IF group (*P* = .046). (Table 3)

**Table 2:**
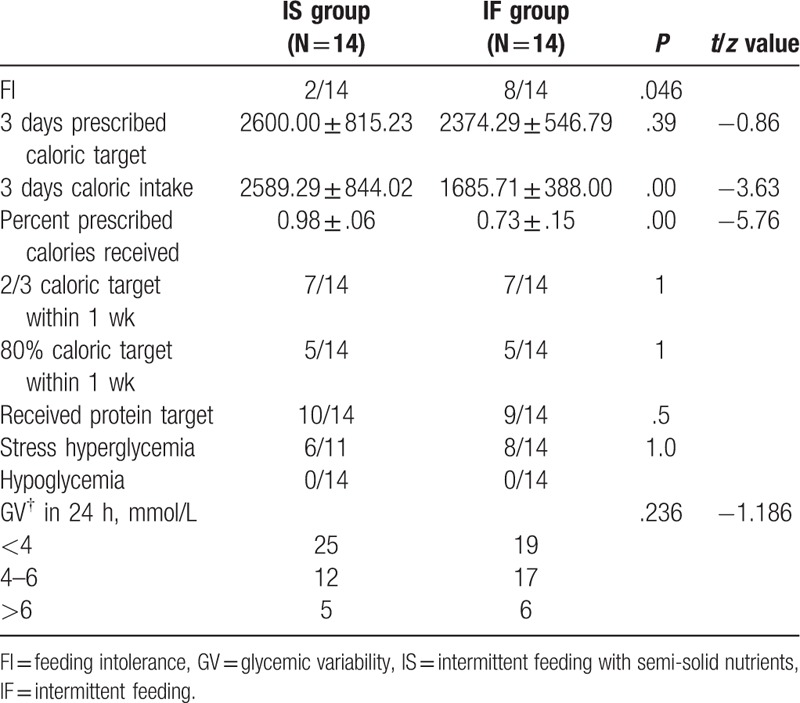
Nutrition delivery and blood sugar.

**Table 3:**
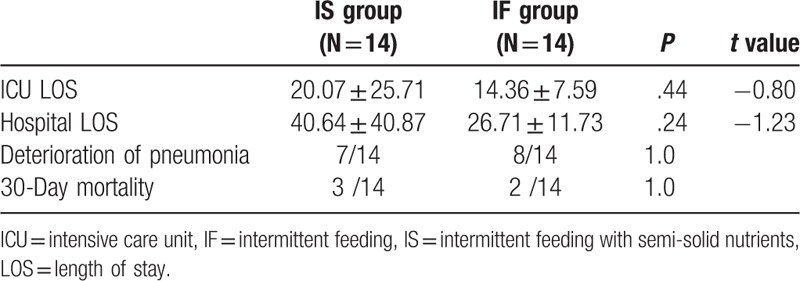
Outcomes.

There were no statistical differences in the secondary outcomes of lung infection, ICU LOS, hospital LOS, in-hospital or 30-day mortality in Table 3. And there were no statistical differences in pharyngeal and endotracheal pepsin levels between the 2 groups suggesting that there were no differences in regurgitation (Table 4).

**Table 4:**
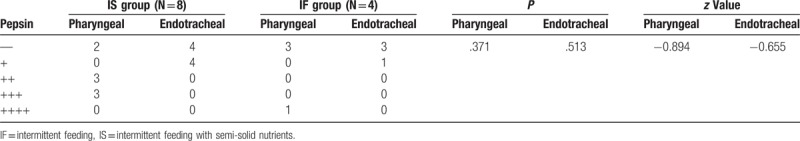
Pepsin.

**Figure 2:**
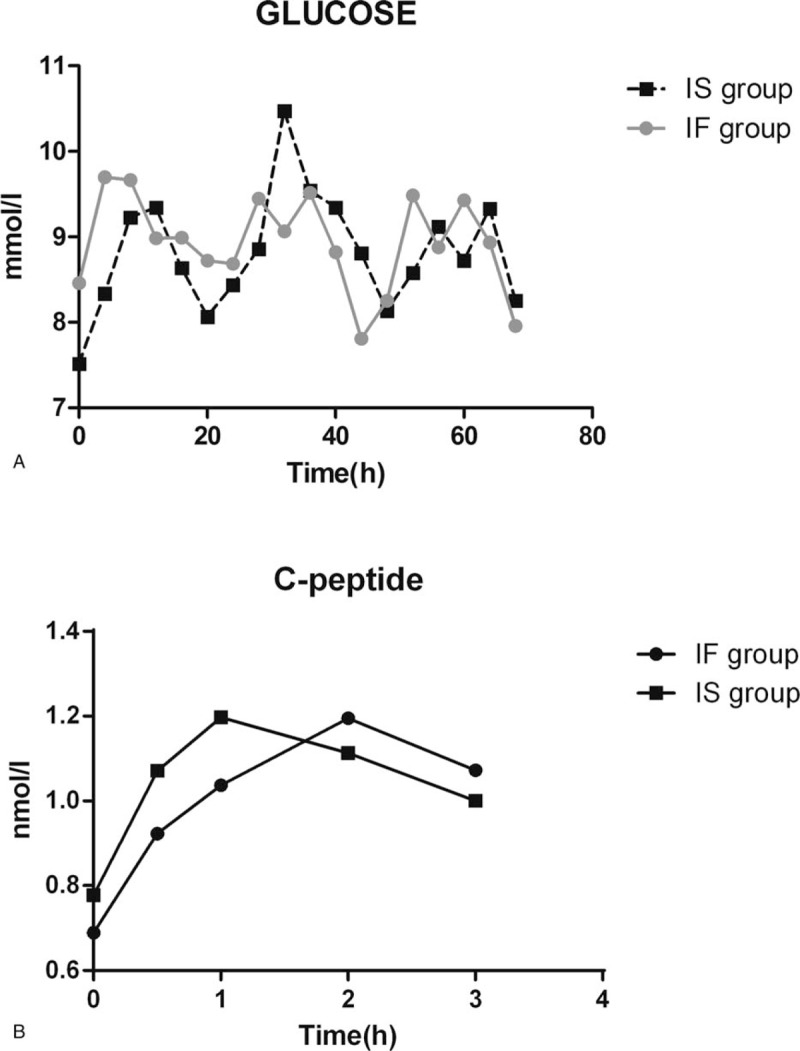
Blood sugar and C-peptide. (A) There were correlation of blood sugar between different time (W = 0.00, *P* < .01). The difference of blood sugar between different time had statistical significance (F = 2.47, *P* = .019). There were no statistical differences between two groups. (F = .015, p = .90). (B) There were correlation of C-peptide between different time (W = .202, *P* < 0.01). The difference of C-peptide between different time had no statistical significance (F = 6.17, *P* < .01). There were no statistical differences between 2 group. (F = .094, *P* = .762). IF = bolus intermittent feeding, IS = bolus intermittent feeding with semi-solid nutrients.

There were no differences in glycemic variability (GV) and stress hyperglycemia for the 72 study hours between 2 groups (Table 2). There was also no incidence of hypoglycemia. As shown in Figure 2A, there was a correlation of blood sugar between different times in each group, but there were no statistical differences between 2 groups, and the correlation of C-peptide was similar to blood sugar between 2 groups (Fig. 2B).

## 4 Discussion

The major finding of this study is that critically ill subjects receiving EN with semi-solid nutrients could increase the efficiency of the EN. And this might be result of improvement of FI, but it does not decrease the LOS in the ICU and in-hospital, lung infection, or 30-day mortality. There were no differences in the regulation of blood glucose.

Underfeeding remains a major challenge in the intensive care unit (ICU), wherein 30% to 60% of patients do not meet their daily estimated protein and energy needs.^[8–10]^ The detrimental effects of malnutrition were most pronounced in critically ill patients.^[11]^ Both baseline malnutrition and worsening nutrition status during hospitalization (regardless of admission nutrition status) were associated with a higher likelihood of complications and costs to the healthcare system.^[10,12]^ Factors, such as delayed gastric emptying, interruptions to feeding owing to fasting for medical interventions, and inadvertent removal of feeding tubes, hinder the provision of adequate nutrition in these patients.^[13–15]^ Meanwhile, as to surgical patients, the consensus of “Enhanced Recovery After Surgery” encouraged patients to mobilize and to eat as soon as possible^[16]^ and the intermittent bolus feeding could shorten the time of feeding that seem to be a better choice to promote the early activity. Previous studies in surgical patients suggest that efforts should focus on maximizing nutrient delivery before and after EN interruptions, rather than on trying to eradicate interruptions.^[17]^ Some researchers suggested encouraging the bolus feeding of EN to achieve daily goals when nutritional feeding was interrupted.^[18]^ In this study, semi-solid nutrients during the first 3 days improved FI and increased the percentage of VR compared with the IF group. It indicated that semi-solid nutrients might effectively increase the efficiency of the EN by improving the FI. There were some possible factors such as the potential longer daily time for feeding, considering the higher incidence of FI, which lead to increased risk of interruption. However, the results showed that the percentage of full target calorie did not increase in IS group, which may be result of only receiving 3 days of semi-solid nutrients.

Previous studies indicated that intermittent infusion had more complications such as diarrhea and regurgitation than continuous.^[4]^ One randomized clinical trial had shown that the thickened liquids reduced aspiration in individuals with dementia and/or Parkinson disease who were easy to aspirate thin liquids.^[5]^ In healthy individuals, pectin increased the viscosity of EN and accelerated gastric emptying.^[19]^ Another study in old adults with a history of aspiration pneumonia or vomiting after PEG found that gastroesophageal reflux was significantly inhibited by semi-solid nutrients. One potential mechanism was considered to be an improvement in the transition from the proximal to distal stomach of semi-solid nutrients.^[20]^ However, other studies also found that semi-solid nutrients may not prevent gastroesophageal reflux.^[21]^ Different from previous studies, our primary end point was FI; this study indicated that the incidence of FI in IS group was lower compared to the IF group.

GV is an independent risk factor which increases mortality, similar to hypoglycemia and diabetes mellitus.^[22]^ Some evidence suggests that continuous infusion may be improving glucose management in enterally fed patients compared to intermittent infusion.^[23]^ Our data indicated that both GV and stress hyperglycemia were well controlled, and there was no hypoglycemia in either group in critically ill subjects without diabetes.

A number of study limitations must be discussed. It is important to note that the present study was performed at a single, tertiary-care center, and only 28 subjects were included; therefore, our results may not be generalizable to all hospitals. Moreover, the time of treatment was only 3 days. We did not find a difference in time to reach caloric target and the percentage of subjects who had up to 80% of the target caloric intake in the ICU. Although we attempted to limit confounding variables including APACHE II score, SOFA score, and GCS in our analyses, but there are likely aspects of individual ICU provider behavior regarding timing, rate, and decision to hold/continue EN infusions that we are unable to control for.

## 5 Conclusions

In this prospective study, we report that the efficiency of the EN was increased by IS, which might be related to the improvement of FI. However, semi-solid nutrients did not decrease the LOS in the ICU and in-hospital, lung infection, or 30-days mortality. There were no differences in blood glucose regulation. Future efforts to increase the sample size and feeding time of semi-solid nutrients will increase the power to detect potential difference in lung infection and mortality between groups.

## Acknowledgments

The authors thank numerous individuals participated in this study, especially the nurse of taking samples.

## Author contributions

**Conceptualization:** kongmiao lu.

**Data curation:** kongmiao lu, Fei Zeng, yi li, cheng chen.

**Formal analysis:** man huang.

**Investigation:** kongmiao lu.

**Methodology:** kongmiao lu.

**Project administration:** kongmiao lu, man huang.

**Software:** kongmiao lu.

**Supervision:** kongmiao lu, Fei Zeng, man huang.

**Writing – original draft:** kongmiao lu.

**Writing – review & editing:** kongmiao lu, man huang.

Author name: 0000-0002-2658-7589.
